# Evaluation of the post-traumatic stress disorder in midwives

**DOI:** 10.1192/j.eurpsy.2025.1965

**Published:** 2025-08-26

**Authors:** I. Sellami, A. Abbes, A. Feki, M. L. Masmoudi, M. Hajjaji, K. Jmal Hammami

**Affiliations:** 1 Occupationnal medecine; 2 Rheumatology, CHU Hedi Chaker, Sfax, Tunisia

## Abstract

**Introduction:**

In addition to the typical risks associated with healthcare professions, being a midwife carries a significant psychological and emotional burden. This responsibility extends not only to the mother’s well-being but also to the newborn’s, making the role particularly vulnerable to psychosocial risks, often driven by high-stress situations.

**Objectives:**

To assess the post-traumatic stress disorder (PTSD) experienced by midwives.

**Methods:**

We conducted a cross-sectional study using a self-administered questionnaire distributed to midwives in the Sfax region. The questionnaire consisted of a first part relating to socio-demographic and professional data and a second part relating to the evaluation of the PTSD in midwives using the Impact of Event Scale (IES).

**Results:**

Our population comprised 74 midwives with an average age of 45.6 ± 10.3 years. Only 21.6% reported engaging in regular physical activity. The midwives worked in both public and private health facilities, with a mean of job tenure of 20.3 ± 10.6 years. A stressful event in their professional life was reported by 68.8% of midwives. The PTSD was detected in 30 midwives (40.5% of the midwifery population surveyed), 8 of whom had severe symptoms. The traumatic events reported by the midwives were related to injury to the newborn, injury to the parturient or working conditions.

We found that the factors related to the PTSD were the number of dependent parents, a history of anxiety, depression or hypothyroidism, and working fixed hours. Physical activity was a protective factor against post-traumatic stress. A statistically significant link was found between severe forms of PTSD and taking leave in the last three months. Binary logistic regression confirmed that while physical activity was protective, anxiety, depression, and hypothyroidism were independent risk factors for PTSD.

**Conclusions:**

PTSD is a common issue among midwives. It should be studied and identified early in at-risk populations to prevent lasting consequences.

**Disclosure of Interest:**

None Declared

